# Genome-Wide Population Structure Analysis and Genetic Diversity Detection of Four Chinese Indigenous Duck Breeds from Fujian Province

**DOI:** 10.3390/ani12172302

**Published:** 2022-09-05

**Authors:** Ruiyi Lin, Jiaquan Li, Yue Yang, Yinhua Yang, Jimin Chen, Fanglu Zhao, Tianfang Xiao

**Affiliations:** College of Animal Sciences (College of Bee Science), Fujian Agriculture and Forestry University, Fuzhou 350002, China

**Keywords:** indigenous duck, population structure, genetic diversity, runs of homozygosity

## Abstract

**Simple Summary:**

The aim of this study was to conduct a genome-wide comparative analysis of four indigenous Chinese duck breeds (Jinding, Liancheng white, Putian black, and Shanma ducks) from Fujian Province, to understand their genetic diversity and population structure. Population parameters showed that the four indigenous breeds were separated groups. Five genomic regions are presented as hotspots of autozygosity among these indigenous duck breeds, with candidate genes involved in muscle growth, pigmentation, and neuroregulation. Genomic information may play a vital role in the improvement of conservation strategies.

**Abstract:**

The assessment of population genetic structure is the basis for understanding the genetic information of indigenous breeds and is important for the protection and management of indigenous breeds. However, the population genetic differentiation of many local breeds still remains unclear. Here, we performed a genome-wide comparative analysis of Jinding, Liancheng white, Putian black, and Shanma ducks based on the genomic sequences using RAD sequencing to understand their population structure and genetic diversity. The population parameters showed that there were obvious genetic differences among the four indigenous breeds, which were separated groups. Among them, Liancheng white and Shanma ducks may come from the same ancestor because the phylogenetic tree forms three tree trunks. In addition, during the runs of homozygosity (ROH), we found that the average inbreeding coefficient of Liancheng white and Putian black ducks was the lowest and the highest, respectively. Five genomic regions were considered to be the hotspots of autozygosity among these indigenous duck breeds, and the candidate genes involved a variety of potential variations, such as muscle growth, pigmentation, and neuroregulation. These findings provide insights into the further improvement and conservation of Fujian duck breeds.

## 1. Introduction

Indigenous duck breeds are an important part of livestock genetic diversity, with rich genetic diversity and breeding potential, and are the basis for breed improvement and the development of new production models [1]. In the past few decades, the breeding progress of indigenous ducks has been slow, and their genetic management has become more difficult due to the preferential use of introduced varieties and indiscriminate hybridization between indigenous breeds [2]. In recent years, more and more attention has been paid to the protection and management of indigenous breeds. The state has effectively protected the genetic resources of local livestock and poultry in China by building indigenous breed-protected farms, protected areas, and waterfowl gene banks [3,4]. However, the protection and development of genetic diversity of local livestock and poultry breeds through the construction of conservation farms are far from enough, and we still know little about these breeds at the genome level [5].

Indigenous ducks have a long history of domestication in China, dating back to 2228 years ago, but their origin has been controversial. A recent report indicated that mallards and Chinese spot-billed ducks were not the pioneers of domestic ducks. They may have come from a ghost wild duck population that was undefined tens of thousands of years ago [6], which will once again cast a mysterious veil over the origin of indigenous ducks. The population structure and domestication characteristics of ducks in eastern China show that spot-billed ducks and mallard ducks were closely related and might contribute to the origin of indigenous ducks [7].

In the process of domestication, human purposeful selection and breeding constantly affect the phenotype of indigenous ducks. Domestic and mallard ducks have greatly different skeletal systems [8]. Jianchang duck is selected in terms of the nervous system, lipid metabolism, and feather color [9]. The phenotypes are different but closely related in eight populations of local ducks in Guangxi [10]. So far, there have been only sporadic reports of genetic studies on indigenous duck populations in China, and the aforementioned studies describe only a small number of indigenous duck breeds. The genomic and genetic information (e.g., population structure) of other indigenous duck breeds remains largely unknown.

**Figure 1:**
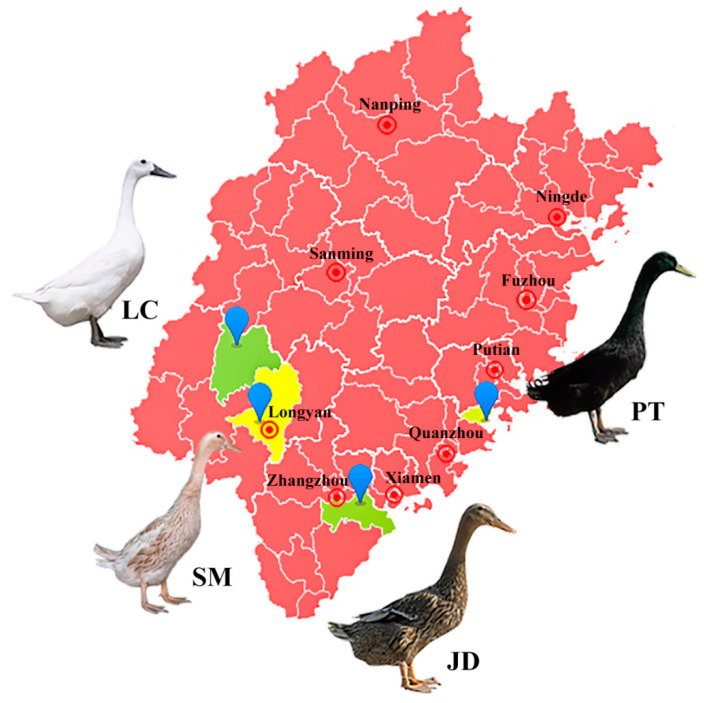
Location of the producing areas of four indigenous duck breeds in Fujian Province. The map was generated by DataMap 6.4 (Global Tone Communication Technology Co. Ltd., Qingdao, China) which is a plug-in for Microsoft Excel 2019 (Microsoft Corporation, Washington, DC, USA). Abbreviations: LC, Liancheng white duck; SM, Shanma duck; JD, Jinding duck; PT, Putian black duck.

Fujian Province is located in southeastern China, adjacent to the Taiwan Strait in the east, and belongs to the subtropics. The complex and diverse geographical and ecological conditions in this province have given birth to a wealth of indigenous duck breeds. To explore the population structure and genetic diversity of these indigenous ducks, we selected four representative breeds (Figure 1). Liancheng white duck (LC) is an ancient breed located in Liancheng County, Longyan City (25°71′ N 116°76′ E). It has been recorded in Chinese history because of its medicinal value, and it is the only all-white feather egg type indigenous duck breed with a black beak and webbed feet. Another breed (Shanma duck [SM]) was mainly distributed in Xinluo District, Longyan City (25°09′ N 117°04′ E) adjacent to Liancheng County, it has many similar characteristics to LC. The average egg production of SM is up to 280 per year. Putian black Duck (PT) originated in Quangang District, Quanzhou City (25°12′ N 118°92′ E). PT duck is the only indigenous breed with black plumage on the whole body, including a black beak and webbed feet. It is widely distributed along the coastline of Fujian Province and deeply liked by local villagers because of its strong adaptability and high laying rate. Jinding duck (JD) is an excellent indigenous duck breed in China, and its main producing area is located in the Longhai District of Zhangzhou City (24°45′ N 117°84′ E). The main characteristic of JD duck is the high proportion of blue-green-shelled eggs. JD, LC, and PT are included in the conservation list of Chinese livestock and poultry genetic resources.

The purpose of this study is to understand the population structure and genetic diversity of Chinese indigenous duck breeds. Here, we described the genetic relationship among four indigenous duck populations in Fujian by principal component, structure, and phylogenetic tree analyses. Run of homozygosity (ROH) is an extension of the above work, which is used to judge valuable genomic information in inbreeding. Our findings can be used to better understand the structural characteristics of the genome among indigenous duck breeds in Fujian Province and to reveal potential selection signs in ROH islands, which is helpful for geneticists and breeders to put forward better conservation strategies and improvement schemes in the future.

## 2. Materials and Methods

### 2.1. Animal Samples and DNA Extraction

A total of 120 blood samples (from LC, PT, JD, and SM duck breeds in China’s Fujian Province, each accounting for 30) were collected in this study. All ducks were raised on a conservation farm at the National Institute of Waterfowl Gene Bank (Shishi, China) and have no shared ancestor for three generations, covering almost all the existing genetic relationships of each breed. Genomic DNA was isolated from all blood samples using the Blood Genomic DNA Extraction Kit (Tiangen Biotech Co., Ltd., Beijing, China) according to the manufacturer’s instructions. Then, DNA concentration and integrity were detected by using a microplate spectrophotometer (Epoch2, BioTek, Winooski, VT, USA) and 1% agarose gel electrophoresis. To avoid the oxidation of duck genomic DNA, it was stored in a −20 °C refrigerator for subsequent reduced-representation sequencing.

### 2.2. RAD Sequencing and Data Sorting

RAD-seq libraries were constructed following a protocol modified from Baird [11]. Briefly, genomic DNA (0.1–1 µg, from either individual or pooled samples) was digested with EcoRI followed by heat inactivation of the enzyme. The digested fragment was end-repaired and ligated with barcoded P1 adapters using T4 ligase. Samples were pooled in proportionate amounts for shearing to an average size of 500 bp. Libraries were size-selected into 300 to 700 bp fragments by running on a 1% agarose gel. Libraries were blunt-end-repaired, and a 3′-adenine overhang was added to each fragment. We added a P2 adapter containing unique Illumina barcodes for each library using the NEBNext adaptor (New England Biolabs, Ipswich, MA). Libraries were amplified through 16 PCR cycles with Phusion High-Fidelity DNA Polymerase and column-purified. Samples were sequenced on an Illumina HiSeq™ 2500 (Illumina, San Diego, CA, USA) using 150 bp paired-end reads. The DNA libraries were sequenced on the Illumina sequencing platform by Gene Denovo Biotechnology Co., Ltd. (Guangzhou, China).

Quality trimming is an essential step to generating high confidence in variant calling. Raw reads would be processed to obtain high-quality clean reads using fastp [12] according to three stringent filtering standards: (1) removing reads with ≥10% unidentified nucleotides (N), (2) removing reads with >50% bases having Phred quality scores of ≤20; and (3) removing reads aligned to the barcode adapter. The PCR-duplicate reads were removed using Picard mark duplicate (v2.18.24). To identify SNPs, the Burrows-Wheeler Aligner was used to align the clean reads from each sample against the *Anas platyrhynchos* genome (GCF_003850225.1) with the settings “mem 4-k32-M,” where -k is the minimum seed length and -M is an option used to mark shorter split alignment hits as secondary alignments [13]. Variant calling was performed for all samples using GATK’s Unified Genotyper (v3.5) [14]. SNPs were filtered using GATK’s Variant Filtration with proper standards (-Window 4, -filter “QD < 2.0 || FS > 60.0 || MQ < 40.0,” -G_filter “GQ < 20”). Minor alleles (maf) and gene deletion rate (geno) are filtered using PLINK (v1.9, Boston, MA, USA), which is set to “—maf 0.05—geno 0.2—allow-extra-chr”, where—allow-extra-chr means to allow additional chromosome input.

### 2.3. Population Structure Analysis

According to the SNP information, we first constructed a distance matrix to represent the evolutionary distance between every two breeds. Then, we cluster-analyzed the samples and inferred the genetic relationship between populations by constantly merging the two nodes with the smallest distance. We used MEGA-X software and neighbor-joining (NJ) methods to construct the phylogenetic tree [15]. Principal component analysis (PCA) was carried out by using the SmartPCA program in EIGENSOFT software [16] based on SNPs. The structure is a clustering algorithm Bayesian model-based analysis. Using ADMIXTURE (v1.3.0) software [17], the population was divided into K subpopulations that obey the Hardy–Weinberger equilibrium. Then, the possibility that the genomic variation of each material originates from the Kth subpopulation was calculated. Thus, the population structure of the sample was inferred. Different SNPs on the genome of the same individual may come from different subpopulations, which is due to the effect of the hybrid process. Linkage disequilibrium (LD) was calculated by using the PLINK software [18]. First, the LD coefficients (r^2^) between two markers in the sample genome were counted, then the LD coefficients were classified according to the distance between markers, and finally, the average r^2^ values in the 200 kb interval were calculated. The r^2^ values of these different intervals were used for linear fitting on R software.

### 2.4. ROH Analysis

ROH is a long and continuous homozygous extension in the genome, which is composed of two identical haplotypes in individuals. The SNP data of 120 samples were merged, and quality control was carried out by PLINK software [19]. The following criteria were used to define the ROH: (1) the minimum number of SNPs that constituted the ROH was set to 100, (2) the minimum SNP density per ROH was set to 1 SNP every 100 kb, (3) the minimum length was set to 1 Mb, (4) two missing SNPs and up to one possible heterozygous genotype were allowed in ROH, (5) the maximum gap between consecutive homozygous SNPs was 1000 kb.

The physical length and number of ROH in each population and the total length of each ROH were calculated by adding the ROH value of the individual. R software was used to draw the ROH statistical chart according to breed classification. The length of the genome covered by ROH was divided by the total duck autosomal genome length covered by the SNP array to estimate individual genome inbreeding coefficients (F_ROH_). The percentage of SNP residing within an ROH was estimated by counting the number of times that each SNP appeared in an ROH and by dividing that number by the total number of animals (120). The SNP region above 30% was selected as the high homozygous region, also called ROH island, and the RCircos package was used to map the ROH distribution on the chromosomes of four indigenous duck populations. To know whether each annotated gene has a biological function, the DAVID database was used to analyze the GO function (cellular component, molecular function, and biological process) and KEGG pathway enrichment of the annotated genes in the ROH island. A comprehensive investigation was carried out according to NCBI and GeneCards websites and existing literature.

## 3. Results

### 3.1. RAD Sequencing of Four Indigenous Duck Populations

Through blood collection and genomic DNA extraction of four indigenous duck breeds, we obtained DNA samples with a concentration of ≥25 ng/μL and OD260/280 values between 1.7 and 2.0. The concentration and purity of these samples can meet the requirements of Illumina sequencing. After sequencing, we obtained the original pairing reads of 389.0 Gb, with each sample averaging 3.2 Gb. Reads with ≥10% unidentified nucleotides and >50% bases having phred quality scores below 20 and reads aligned to the barcode adapter were discarded. Finally, clean paired reads of 352.2 Gb were obtained after quality filtering. The sequence analysis results showed that the quality of Q20 ranged between 94.23% and 97.70% with an average value of 96.29%. The quality of Q30 ranged from 88.52% to 93.98% with an average value of 91.36%. By referring to genome alignment, we detected an average of about 1.4 million SNP loci in four indigenous breeds.

### 3.2. Population Genetic Structure of Four Indigenous Duck Breeds

**Figure 2:**
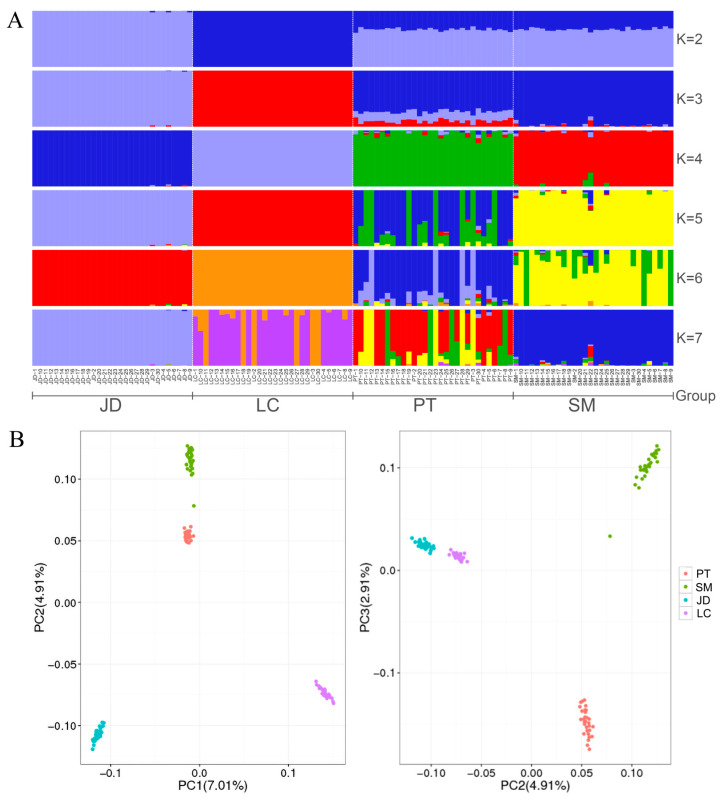
Analysis of population variation and structure analysis of duck breeds. (**A**): Population structure of indigenous ducks. (**B**): Principal component analysis (PCA).

To estimate different ancestor ratios, we used the ADMIXTURE analysis software to determine the optimal clustering and analyze the population structure based on the results of the cross-validation (CV) error rate. Our CV error results showed that the optimal number of clusters = 3 (Figure S1). The analysis of population structure assumes that there are K ancestral populations (Figure 2A). When K = 2, LC was first isolated. Interestingly, PT and SM showed mestizos, with an average of 66% and 34% of their lineages assigned to JD and LC breeds, indicating that they may be JD × LC hybrid populations. When K = 4, we can see four distinct and clear groups, indicating that these four breeds have a unique genetic structure, which is the same as the sample information collected, but also consistent with the expected results of this study. Of note, PT and SM populations have three other lineages except themselves, indicating that they may have slight gene communication in modern times. In addition, PTs and SMs showed more mixed blood at K = 6. These results are not surprising because hybridization is very common in birds [20]. However, JDs and LCs still have a single ancestor, indicating that they have a simple pedigree, which is likely attributable to inbreeding or population founder effects.

To explore the genetic background of four indigenous duck breeds, PCA was carried out based on genome-wide SNP data. The results showed (Figure 2B) that PCA demonstrated a clear genetic structure with the samples from each variety clustering together. In the first component of the PCA, LCs and JDs were divided into different clusters, which were different from PTs and SMs, indicating that PTs and SMs may be more similar in history. In the second component, the four clusters are independent. And in the third component, PTs and SMs were separated by JDs and LCs. In addition, we noticed that a vagrant of SM showed a close background relationship with PT, and this individual had a high proportion of PT pedigree in the population structure, indicating that its parents may have participated in the mating plan of PT.

**Figure 3:**
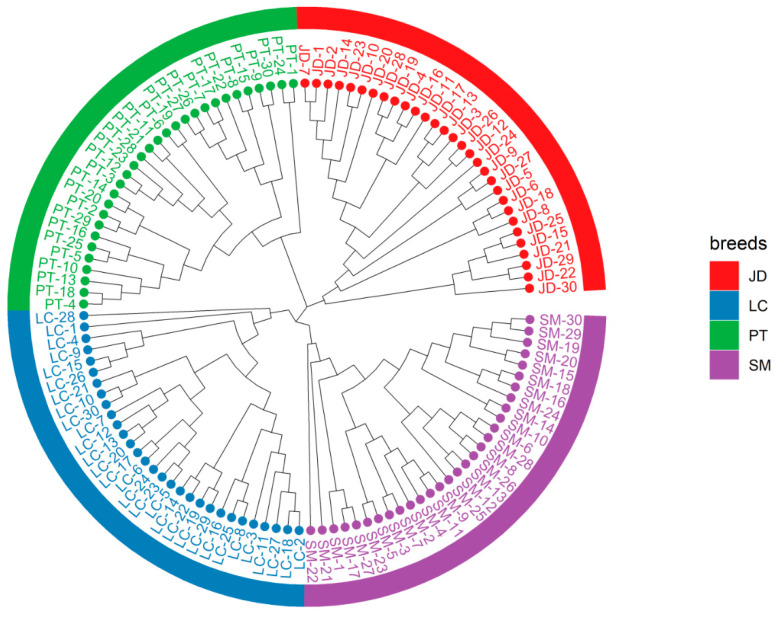
Phylogenetic trees showing the genetic structure of the 120 indigenous ducks individuals.

The phylogenetic tree is very important for understanding the genetic relationship of the population. We used the Kimura two-parameter model to repeat sampling 1000 times to form a more reliable phylogenetic tree. It gathers the samples with high sequence similarity together to obtain the NJ tree. The bootstrap values of the NJ tree topology are all above 75%, indicating that the phylogenetic tree we constructed has high credibility. The results showed (Figure 3) that all individuals from the same breed were clustered into a subbranch, indicating that there was obvious genetic differentiation among these indigenous breeds. The NJ tree finally converged into three lines, and JD and PT formed separate branches. Moreover, LC and SM were finally grouped into the same group, which means that they have a close relationship and may come from a common ancestor. Our results realize the accurate division of the evolutionary relationship of different duck populations.

### 3.3. Evaluation of Genetic Diversity by Linkage Disequilibrium Trend

**Figure 4:**
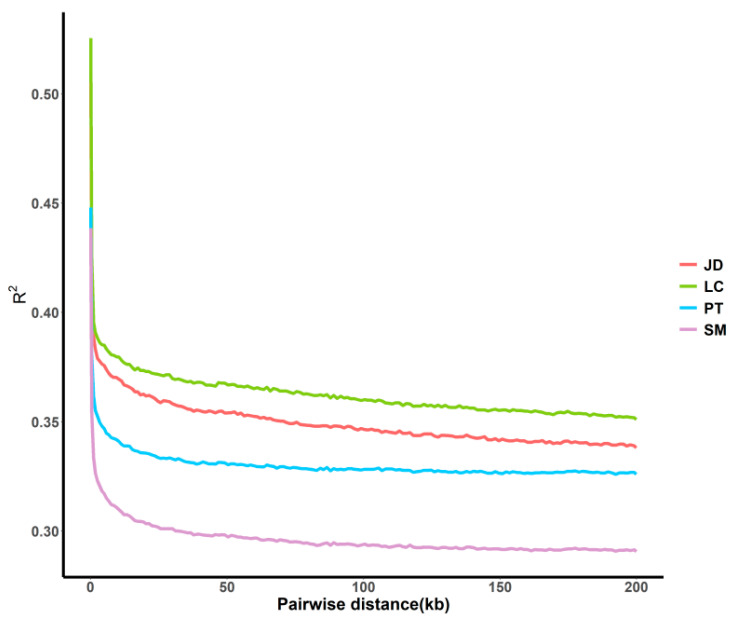
LD attenuation diagrams of duck populations.

To obtain a more specific understanding of the genomic characteristics within the population, we used r^2^ as the LD coefficient to measure the degree of linkage between loci. The LD linear fitting results (Figure 4) showed that the degree of LD and the attenuation degree of locus association were different among different populations and within the same SNP spacing, in the order of SM > PT > JD > LC. Obviously, SM had the lowest LD score, whereas LC had the highest score. In total, LD decreased with the increase of physical distance between markers.

### 3.4. Runs of Homozygosity Detection and Analysis

**Figure 5:**
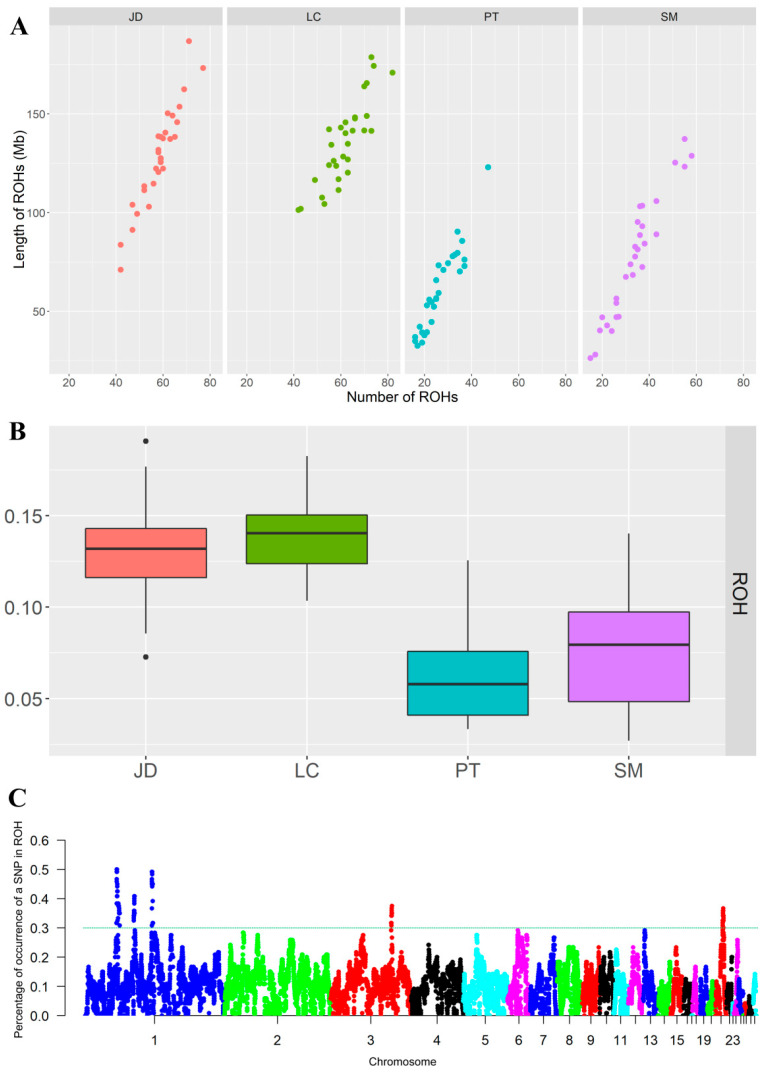
Genome-wide scan for ROH. (**A**): Number of ROH per breed (X-axis) and the total ROH length of each animal (Y-axis). (**B**): Box plot of the inbreeding coefficients inferred from ROH (F_ROH_) for duck breeds. (**C**): Manhattan plot of the incidence of each SNP in the runs of homozygosity among the duck breeds.

A total of 5373 ROH were detected in four indigenous duck breeds, including 1751 ROH (32.59%) in JD, 1853 ROH (34.49%) in LC, 788 ROH (14.67%) in PT, and 981 ROH (18.26%) in SM. The number and total length of ROH in the four groups of ducks are shown in Figure 5A, and the long ROH is generally considered to be a sign of inbreeding. The number and total length of ROH of LC and JD were larger than those of PT and SM. The total length of ROH of most LC and JD was above 100 Mb (mean value was 128.38 and 135.75, respectively), whereas that of PT and SM was below 100 Mb (mean value was 98.64 and 76.93, respectively).

ROH data were used to evaluate the average inbreeding coefficient of different duck populations (Figure 5B). LC had the highest average inbreeding coefficient (F_ROH_ = 0.14), followed by JD (F_ROH_ = 0.13), SM (F_ROH_ = 0.08), and PT (F_ROH_ = 0.06). At the individual level, the animal with the highest genomic inbreeding coefficient appeared in the JD population (ROH_max_ = 0.191), and that with the lowest genomic inbreeding coefficient appeared in the SM population (ROH_min_ = 0.027). The above results suggest that JD and LC may have artificially directed inbreeding.

Nothnagel et al. first defined the genomic regions associated with inbreeding as ROH islands, that is, overlapping homozygosity regions are highly shared among individuals belonging to the same population [21]. To identify high homozygosity regions within the population, we selected 0.3 as the threshold, which will indicate the possible existence of ROH islands in the genome (Figure 5C). Table 1 shows the Chromosome location of the homozygosity region of these genomes, the number of SNPs owned, the starting and ending sites, and the physical length of the region. A total of five ROH islands were identified: three regions on Chr1 (45.62–50.13, 70.79–72.14, and 97.15–98.97 Mb), one region on Chr3 (88.62–89.67 Mb), and one region on Chr21 (10.21–11.27 Mb). To explore the characteristics of these ROH islands, we performed GO and KEGG analysis of 64 genes annotated on the island to determine whether indigenous ducks are undergoing potential variation in selection. The results are shown in Figures S2 and S3.

## 4. Discussion

The assessment of population genetic structure and diversity is the basis for understanding the genetic information of indigenous breeds, and it is important for the protection and management of local breeds [22]. In recent years, there have been sporadic reports on the genetic diversity and population structure of some indigenous duck populations in China based on microsatellite markers [23], mitochondrial markers [24], and whole-genome resequencing [10]. Here, we used a genome-wide restriction site-associated DNA sequencing (RAD-seq) technique to describe the genetic diversity and population structure of four Fujian indigenous duck breeds based on genome-based SNP markers. The results of structural analysis and PCA were basically consistent with the phylogenetic tree, which confirmed that the four indigenous breeds were independent ecological populations. The results of LD analysis and ROH analysis showed that PT and SM were rich in genetic diversity.

In this study, we sequenced 120 ducks. Structure analysis, PCA, and phylogenetic tree revealed the population structure of four indigenous duck breeds in Fujian Province. The results were basically consistent with our expectations. Structure and PCA analysis clearly divided them into four groups, and the phylogenetic tree also formed four subbranches, indicating that these breeds had obviously differentiated. In addition to the long history of breeding, the reason for differentiation may also be attributed to geographical isolation. Although the main producing areas of these indigenous breeds are relatively close on the map, Fujian Province has complex topography, with many hills, mountains, and rivers. SM and PT gathered close to each other on the PCA map and mixed each other’s pedigree in the structural analysis, indicating that there was a close relationship between them. In addition, we constructed the evolutionary trees of these four indigenous duck breeds and tried to infer the phylogenetic relationship between them. The results showed that the two breeds belong to the same big branch, different from the previous research results [25]. SM and LC are sister branches in the tree, showing a high homologous relationship. Moreover, in structural analysis, SM has a certain proportion of the ancestors of LC in K = 2. This may be because we chose RAD-seq with a higher resolution than in previous studies. In fact, the main producing areas of SM and LC have the closest geographical distance. In other words, these results support the genomic similarity between the two populations, and SM may be formed by the evolution of LC through migration and selection. Taken together, these four indigenous breeds may have been domesticated for a long time and differentiated into independent populations.

The differences in LD in the current study may be attributed to different selection and evolutionary forces in the process of variety formation [26]. The results of our analysis show that the degree of linkage of SM is lower and the heterozygosity is higher, which may be due to the late start of breeding work and less selection in this indigenous breed.

ROH analysis has become a standard method for studying inbreeding and detecting the selection characteristics of animal populations [27]. McQuillan et al. first defined the genomic inbreeding coefficient (F_ROH_) based on ROH and indicated that high F_ROH_ usually represents inbreeding [28]. ROH analysis is considered to be one of the most effective methods to detect inbreeding at present [29,30]. In general, variants experiencing directional selection usually show strong LD and long-range haplotype homozygosity as the frequencies of the variants rapidly increase [31]. In this study, the analysis results of LC and JD likely run in the same groove as above, which indicates that their high inbreeding may be due to artificial selection. This result also explained the simple lineage composition in structural analysis and proved that inbreeding in history had a great impact on the genomes of JDs and LCs. The process of accepting the selection of breeds is also accompanied by the decline of genetic diversity. In future breeding plans, we need to pay attention to the fact that cousins of the same population should enjoy a lower priority in pairing, which helps to reduce the loss of genetic diversity.

The length and quantity of ROH can infer the historical information of varieties at the individual level [32]. However, inbreeding leads to uneven distribution of ROH in the genome, and the number and size of ROH on each chromosome are different. Interestingly, ROH, which is located in a specific chromosome region and shared among several individuals, can reflect potential signs of selection [33], which Ablondi et al. believe is the result of directional selection driving forces [34]. In this study, we found five ROH islands, which are located on chromosomes 1, 3, and 21. Through enrichment analysis, we found the genes of pathway mapping, which can be used to define a selection for economic traits and environmental adaptation of breeds [35]. Several of these genes are worth mentioning because they are associated with several important economic traits. *GNAS* connects to the Gap junction pathway, which encodes Gsα, XLαs, NESP55, and A/B protein. It has been identified as a candidate gene related to skeletal muscle growth and development in Chinese native pig breeds [36], and it may regulate daily gain, feed conversion rate, carcass traits, and other important economic traits [37]. Imumorin et al. also verified this point in cattle [38]. In addition, *GNAS* is an important regulatory gene of pigment cells. *EDN3* is a gene that encodes endothelins (EDNs), which participate in the regulation of some physiological processes, such as cardiovascular development and function, pigmentation, and plumage in birds [39]. Embryonic development is regulated by *EDN3–EDNRB2* signals, which are closely related to melanin deposition in duck epidermis [40] and feather patterns [41], consistent with the results of our previous study [42]. Coincidentally, the gene was also selected in the Guangxi chicken population [43]. *GNAS* and *EDN3* genes were included by the European Society for Pigment Cell Research (http://www.espcr.org/micemut, accessed on 29 May 2022), suggesting that they may play an important role in coat color formation in indigenous ducks. Similar to the former, the *VAPB* gene also regulates the skin color of birds [44]. Hou et al. used GWAS and F_ST_ methods to mine the genes (*EDN3* and *VAPB*) that are essential for the development of melanocytes [45]. These results may explain the occurrence of interesting phenotypes in indigenous duck breeds. SLITRK1 and SLITRK6 are members of the Slitrk family. They are usually found in the central nervous system and are highly expressed transmembrane proteins in the central nervous system. Specifically, the *SLITRK1* gene is preferentially located in the excitatory synapse of hippocampal neurons. It has the potential to promote presynaptic differentiation of excitatory and inhibitory synapses [46]. The *SLITRK6* gene is related to the neural regulation of vision and hearing and plays an important role in the development of normal hearing and vision [47]. The enrichment of the above genes is significant (*p* < 0.05), which may imply that the important traits such as growth, development, and appearance color of these ducks have endured strong artificial selection. Such gene variation events have different selectivity in different breeding practices, which may be beneficial to heterosis.

## 5. Conclusions

In this study, the population structure and genetic diversity of four indigenous duck breeds in Fujian Province were analyzed in detail from the whole genome perspective. The characteristics of these breeds between and within populations were evaluated by using genomic data through a variety of methods. Our comprehensive analysis showed that the four breeds are all independent populations, and muscle growth and pigment deposition are the variation direction of artificial selection at present. Therefore, the population structure and candidate genes described here may be used to clarify the differentiation of Fujian indigenous ducks and to improve their breeding. Our findings will help to better manage these unique genetic resources and improve mating management, which is essential for protecting genetic diversity and promoting the development of indigenous breeds.

## Figures and Tables

**Table 1:** Genomic regions of extended homozygosity (ROH islands) identified in the 4 indigenous duck breeds.

Chr	Num of SNPs	Start	End	Length (bp)	Genes
1	108	45626183	50131802	4505620	LOC113839685, LOC106014954, LOC113839687, LOC113839688, LOC113839689, SLITRK1, LOC113839691, LOC113844965, LOC113839693, SLITRK6, LOC106017061, LOC113839694, SLITRK5, LOC110354298, MRPS12, LOC106019638, LOC110352518
1	51	70797389	72148337	1350949	LOC101791573, LOC113840661, LOC106016827, LOC106016828, NLGN4X
1	66	97150638	98972361	1821724	GABPA, ATP5PF, JAM2, MRPL39, LOC106014525, LOC106014508, LOC106014510, LOC110351253, LOC106014431, LOC113842349, NCAM2
3	66	88621432	89675961	1054530	LOC106016669
21	50	10214904	11278269	1063366	LOC113845753, LOC110353825, LOC106019012, C21H20orf85, ANKRD60, LOC101805171, RAB22A, *VAPB*, APCDD1L, STX16, NPEPL1, LOC113839645, LOC106018983, *GNAS*, LOC101792091, LOC106018981, LOC113839646, NELFCD, CTSZ, TUBB1, ATP5F1E, LOC106018967, PRELID3B, ZNF831, *EDN3*, PHACTR3, SYCP2, LOC101790963, FAM217B, LOC113839657

## Data Availability

The dataset supporting the conclusions of this article is available with links to BioProject accession number PRJNA862275 (https://www.ncbi.nlm.nih.gov/bioproject/PRJNA862275, accessed on 30 August 2022).

## Supplementary Materials

The following supporting information can be downloaded at: https://www.mdpi.com/article/10.3390/ani12172302/s1, Figure S1: Cross-validation error line charts of duck breeds; Figure S2: GO enrichment analysis of candidate genes in the ROH islands; Figure S3: KEGG pathway enrichment analysis of candidate genes in the ROH islands.
