# Supplementary material for: Genome-Wide Population Structure Analysis and Genetic Diversity Detection of Four Chinese Indigenous Duck Breeds from Fujian Province
Source: Animals (Basel). 2022 Sep 5;12(17):2302. doi: 10.3390/ani12172302 (PMC9454422; doi:10.3390/ani12172302)
Supplement: Supplementary file 1 [file animals-12-02302-s001.zip › animals-1846789-supplementary/Supplementary/animals-1846789-supplementary.pdf]

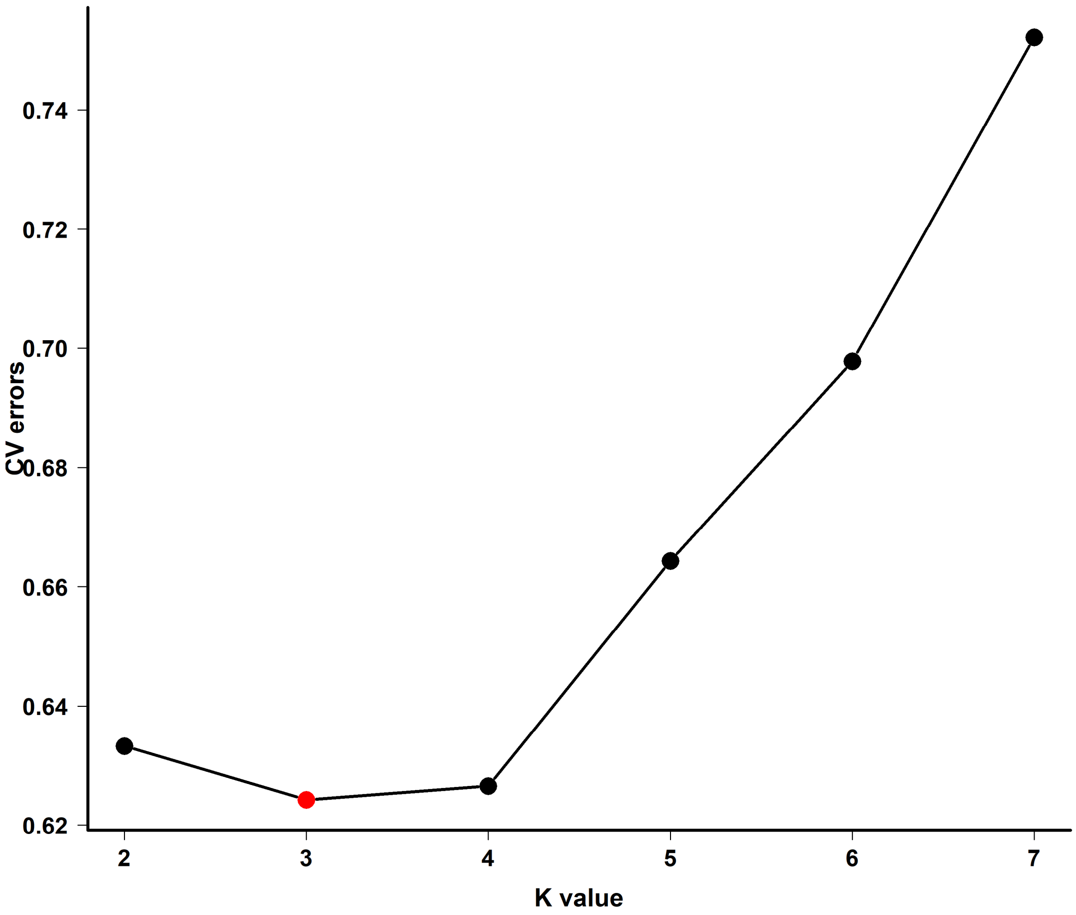

Figure S1. Cross-validation error line charts of duck breeds. The optimal number of assumed ancestors was three (K=3), at which point the error reached the lowest level.

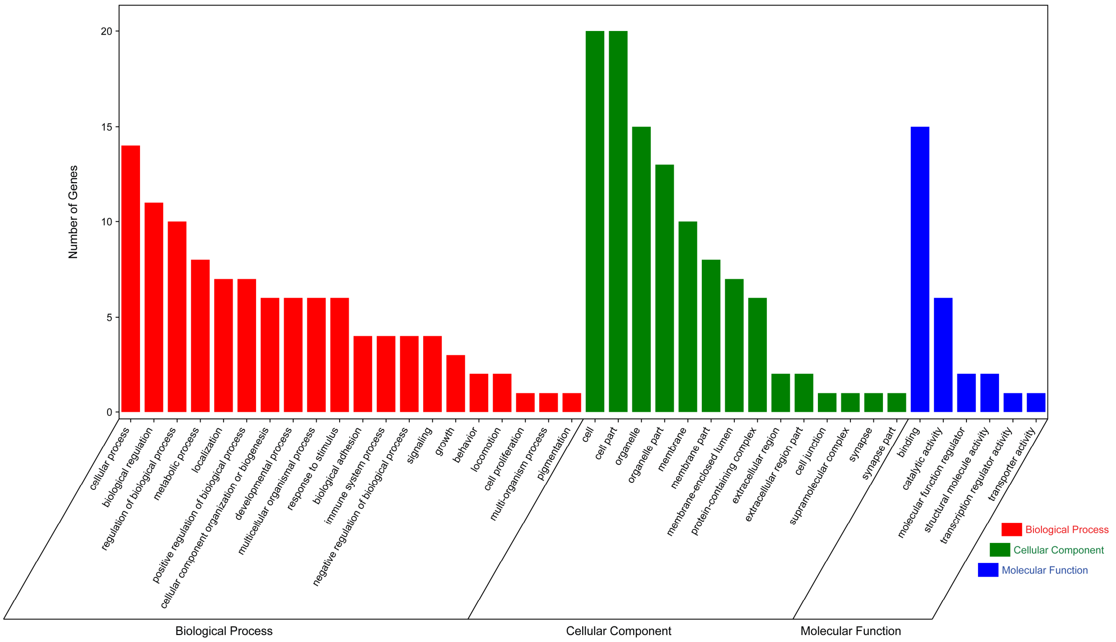

Figure S2. GO enrichment analysis of candidate genes in the ROH islands. The red, green, and

blue parts represent biological process, cellular component, and molecular function, respectively.

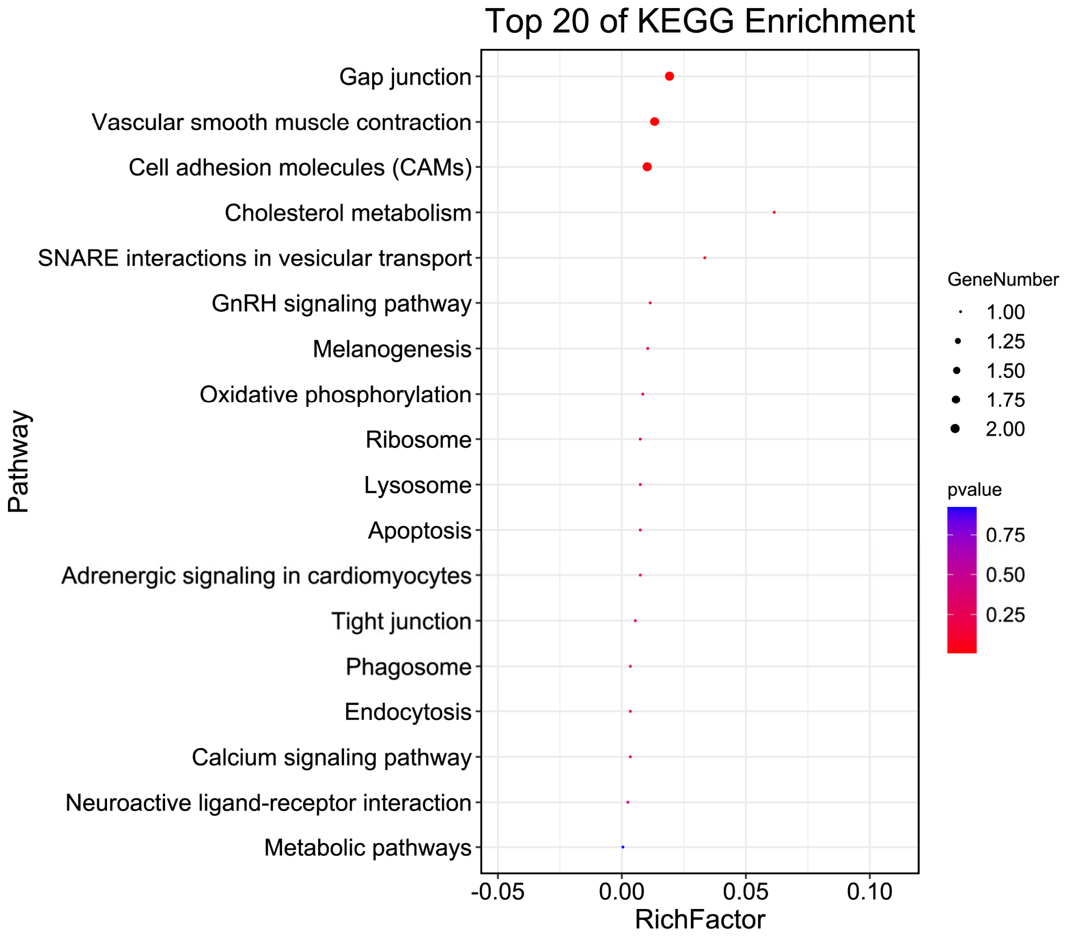

Figure S3. KEGG pathway enrichment analysis of candidate genes in the ROH islands.
